# Colposcopy telemedicine: live versus static swede score and accuracy in detecting CIN2+, a cross-sectional pilot study

**DOI:** 10.1186/s12905-018-0569-1

**Published:** 2018-06-11

**Authors:** Katayoun Taghavi, Dipanwita Banerjee, Ranajit Mandal, Helena Kopp Kallner, Malin Thorsell, Therese Friis, Ljiljana Kocoska-Maras, Björn Strander, Albert Singer, Elisabeth Wikström

**Affiliations:** 1 Institute of Social and Preventive Medicine (ISPM), University of Bern, Bern, Switzerland; 2 Department of Gynecologic Oncology, Chittaranjan National Cancer Institute, Kolkata, India; 3 Department of Clinical Sciences, Danderyd Hospital, Karolinska Institutet, Stockholm, Sweden; 4 Akademiska sjukhuset, Uppsala University Hospital, Uppsala, Sweden; 5 Department of Women’s and Children’s Health, Uppsala University, Uppsala, Sweden; 6 Department of Clinical Sciences, Sahlgrenska academy, University of Gothenburg, Gothenburg, Sweden; 7 University College Hospital, London, UK

**Keywords:** Colposcopy telemedicine, Cervical screening, Low-resource settings, Gynocular, Mobile colposcope

## Abstract

**Background:**

This cross-sectional pilot study evaluates diagnostic accuracy of live colposcopy versus static image Swede-score evaluation for detecting significant precancerous cervical lesions greater than, or equal to grade 2 severity (CIN2+).

**Methods:**

VIA or HrHPV positive women were examined using a mobile colposcope, in a rural clinic in Kolkata, India. Live versus static Swede-score colposcopy assessments were made independently. All assessments were by gynecologists, junior or expert. Static image assessors were blinded to live scoring, patient information and final histopathology result. Primary outcome was the ability to detect CIN2+ lesions verified by directed biopsies. Diagnostic accuracy was calculated for live versus static Swede-score in detecting CIN2+ lesions, as well as for interclass correlation.

**Results:**

495 images from 94 VIA positive women were evaluated in this study. Thirteen women (13.9%) had CIN2+ on biopsy. No significant difference was found in the detection of CIN2+ lesions between live and static assessors (area under curve = 0.69 versus 0.71, *p* = 0.63). A Swede-score of 4+, had a sensitivity of 76.9% (95% CI 46.2–95.0%) and 84.6% (95% CI 54.6–98.1%), for live- and static-image assessment respectively. The corresponding positive predictive values were found to be 90.9% (95% CI 75.7–98.1%) and 92.6% (95% CI 75.7–99.1%). The interclass correlation was good (kappa statistic = 0.60) for the senior static assessors.

**Conclusions:**

Swede-score evaluation of static colposcopy images was found to reliably detect CIN2+ lesions in this study. Larger studies are needed to further develop the colposcopy telemedicine concept which may offer reliable guidance in management where direct specialist input is not available.

**Trial registration:**

Ethical approval of the study was obtained by the Chittaranjan National Cancer Institute (CNCI) Human Research Ethics Committee (4.311/27/2014). The trial was retrospectively registered in the Clinical Trails Registry of India CTRI/2018/03/012470.

**Electronic supplementary material:**

The online version of this article (10.1186/s12905-018-0569-1) contains supplementary material, which is available to authorized users.

## Background

Today, cervical cancer is largely preventable through regular screening, and countries with established cervical screening programs have seen up to an 80% reduction in the incidence of disease [1, 2]. Worldwide, almost 9 out of 10 deaths from cervical cancer occur in low- and middle-income countries (LMIC) [3]. This is largely due to a lack of organised screening programs, shortage of specially trained clinicians, as well as the high costs and immobility of diagnostic equipment [4, 5]. The problem is also present in European countries where there is a lack of organised screening programs or reduced access to screening services [1, 6].

The evolution of telecommunication technologies has given rise to the field of telemedicine, which allows specialised input regardless of geographical location. While not specific to cervical cancer screening, previous studies have found a positive association between telemedicine and increased access to specialized care, decreased costs to patient, reduced treatment costs at earlier stage of disease, and overall increased socioeconomic returns [7]. With increasing access to internet and mobile phone connectivity, telemedicine offers potential solutions for improving access and quality of cervical screening for women in low-resource settings as well as those who have reduced access to screening services due to geographical station [8–12].

Globally, cervical cancer screening and examination is performed by nurses, midwives and colposcopy specialists with varying accuracy. Supervision and support by senior clinicians is often difficult to obtain [4]. A single visit approach using Visual Inspection with Acetic acid (VIA) has been shown to have benefits in low-resource settings [13–16]. This approach is recommended by the World Health Organization (WHO) when there is no other screening option, as it is the cheapest and most feasible method of screening in some countries, ensuring a strong connection between screening and treatment [17]. However, these programs often lack quality controls and the efficacy of screening may be further undermined by inter-observer variation due to subjectivity of assessments and variable accuracy, all of which are inherent in visual assessments [4, 18, 19]. Telemedicine has the potential to overcome the lack of on-site supervision [4], and increase the accuracy of assessments [11, 12]. Investigating the accuracy of static image colposcopy images by specialists is a fundamental step in determining the feasibility of colposcopy telemedicine.

This pilot study evaluates the diagnostic accuracy of live colposcopy Swede score evaluation versus static image Swede score evaluation for detecting cervical intra-epithelial neoplastic lesions greater than, or equal to grade 2 severity (CIN2+). We investigate the differences in accuracy between live specialist assessment and static image assessment using the Gynocular colposcope and its mobile phone application.

## Methods

This cross-sectional community-based study was conducted in cervical screening camps, covering the rural parts of West Bengal, in Kolkata, India, between April 2014 and April 2016 (Trial registration: CTRI/2018/03/012470). Ethical approval of the study was obtained by the Chittaranjan National Cancer Institute (CNCI) Human Research Ethics Committee (4.311/27/2014). Oral and written consent was obtained before inclusion in the study.

Non-pregnant, previously unscreened women between 30 and 60 years of age with uteri were eligible for the study and invited to participate consecutively. All women received both VIA (performed in accordance with the WHO guidelines [20]) and high-risk oncogenic human papillomavirus (HR-HPV) testing (Hybrid Capture 2, QIAGEN™, Gaithersburg, USA [21]). Those who were either VIA or HR-HPV positive had a colposcopy examination on-site by a senior clinician using the Gynocular (Gynius AB, Stockholm, Sweden) [22]. The Gynocular is a hand held, battery operated, monocular colposcope with green light filter and optical zoom capacity between 4-14X. A total of 94 screen-positive women were recruited for the study. Standard colposcopic examination was performed, including visualisation of the vagina, vulva and cervix following insertion of a speculum, examination of cervical vessel patterns using the red-free mode (or green filter), application of 5% acetic acid for 1 min and finally assessment following application with Lugol’s iodine. The examination was completed with at least one biopsy from the most severe lesion. When no lesion was seen, a biopsy was taken randomly from the squamocolumnar junction. The findings of the live examination were documented using the parameters of the Swede score, which includes measurements for acetowhiteness, appearance of margins, vessels, lesion size, and iodine staining (Table 1) [23]. Each parameter is scored between zero and two. Treatment is based on the summed total. A treatment threshold of 5 was used in this study [24].

**Table 1:** Swede score and description of scoring [17]

Score	0	1	2	Total
Acetowhite uptake	Nil or transparent	Shady, milky (not transparent not opaque)	Distinct, opague white	
Margins surface	Diffuse	Sharp but Irregular, jagged, “geographical” satellites	Sharp and even, difference in surface level such as “cuffing”	
Vessels	Fine, regular	Absent	Coarse or atypical	
Lesion size	< 5 mm	5–15 or or 2 quadrants	> 15mm or spanning 3–4 quadrants or endocervically undefined	
Iodine staining	Brown	Faintly or patchy yellow	Distinct yellow	
Total				/10

Julie Bowring, Bjorn Strander, Martin Young, Heather Evans, Patrick Walker, The Swede Score: Evaluation of a Scoring System Designed to Improve the Predictive Value of Colposcopy, Table 1, Journal of Lower Genital Tract Disease, vol 14, issue 4, pages 301–305

https://journals.lww.com/jlgtd/Abstract/2010/10000/The_Swede_Score__Evaluation_of_a_Scoring_System.5.aspx promotional and commercial use of the material in print, digital or mobile device format is prohibited without the permission from the publisher Wolters Kluwer. Please contact permissions@lww.com for further information. License to reprint Swede Score Model is included as an additional document to this paper (Additional file 1)

**Fig. 1:**
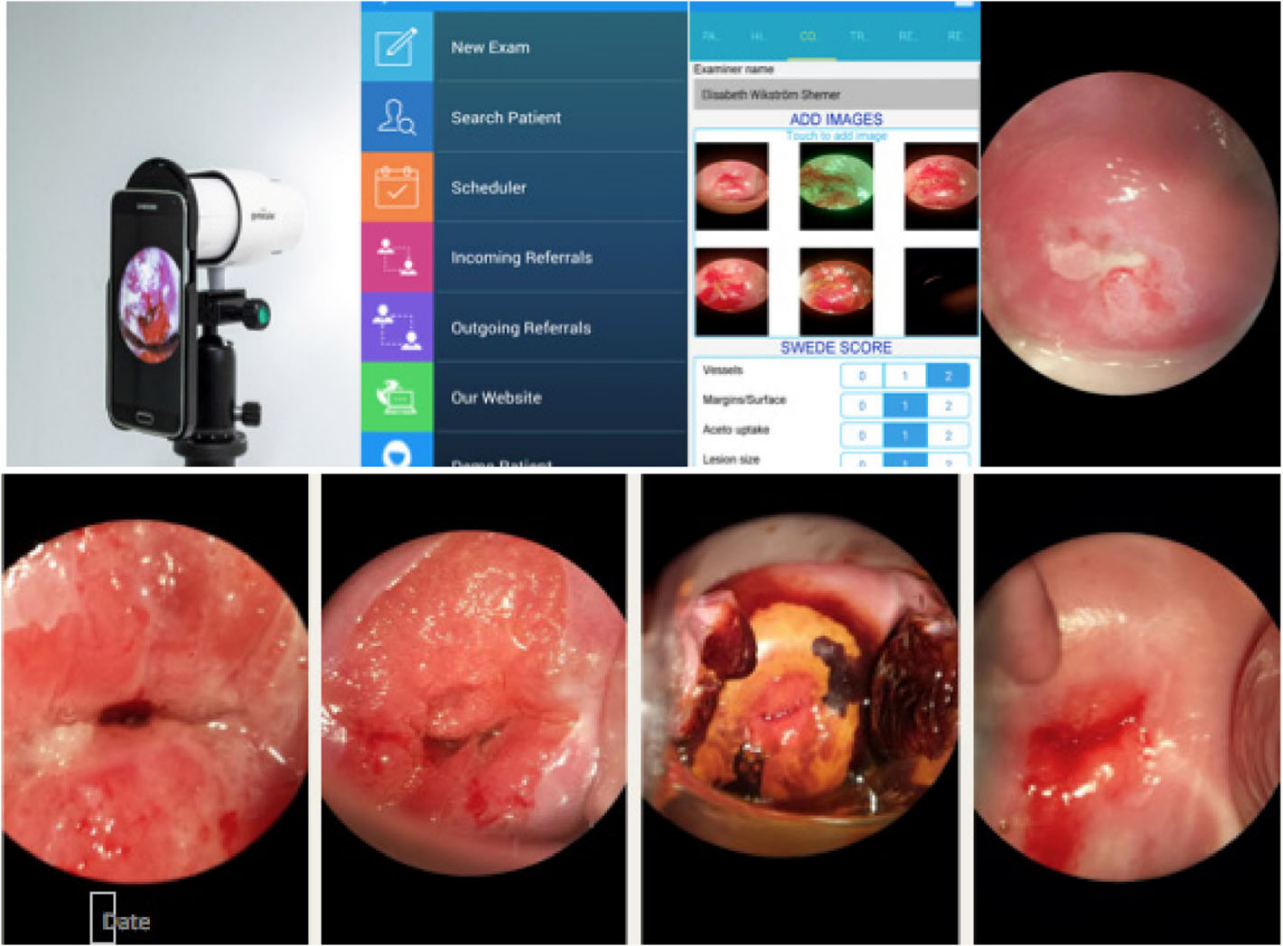
Colposcopy telemedicine equipment. From left to right - Gynocular device, telemedicine smartphone based patient record system, clinical examination and Swede score tool. Below - images obtained from the unit

Colposcopic images were captured thorough the Gynocular colposcope using a Samsung Galaxy S3 mobile (Samsung Electronics, Seoul, South Korea). The Samsung S3 mobile includes an 8 megapixel camera with a zoom function (Fig. 1). Up to six photographs were used for each patient. Timing of the pictures, following these steps, was at the discretion of the colposcopist and not specified in the study protocol. All photos were taken before biopsies were obtained. For each patient, there were no two pictures that were the same. However, some steps of the colposcopy examination may not have been captured, and at some images were similar, but using both low and high magnification. All images pertaining to each patient were assessed to estimate the Swede score.

Assessors included one live colposcopist, a senior gynecology oncologist and six international colposcopists (four senior, two junior). The group of colposcopists were residing in India, Sweden, United Kingdom and Switzerland. The static assessors each reviewed the images from every participant. No additional clinical information was provided.

All women had one or more cervical biopsy evaluated according to clinical routine. In cases where there was no visible lesion, a random biopsy was taken from the squamocolumnar junction and evaluated. Diagnostic accuracy was calculated for live versus static Swede score impression in detecting CIN2+ lesions. CIN2 is a well-established threshold for treatment in cervical screening due to the increased risk of progression from this stage and beyond [25]. This was a pre-specified threshold and is widely accepted for the assessment of diagnostic accuracy in cervical cancer screening studies [13, 20]. Interclass correlation was also assessed.

### Statistics analysis

Descriptive statistical methods were used to report patient baseline demographic and clinical characteristics using percentages and standard deviations (SD). Histological biopsy findings of CIN2+ was used as the reference standard in the study. We calculated test accuracy (sensitivity, specificity, positive predictive value, and negative predictive value) with corresponding 95% Confidence Intervals (CIs) for live colposcopy Swede score and corresponding static colposcopy Swede scores to detect CIN 2+. The receiver operator curves (ROC) for Swede scores were plotted as sensitivity against 1-specificity. To test reliability of the test and the level of agreement between live and static image colposcopy assessment, we calculated the percentage agreement and the weighted kappa (ĸ) statistic [26]. The statistical software used to analyse results was R version 3.2.5.

## Results

In this study, 495 images from 94 VIA positive women were evaluated. The average age of women was 37 years. The incidence of CIN2+ was 13.8%, as expected in an unscreened general population [10]. This was mostly detectable by VIA (*n* = 12/13 CIN2+ lesions were VIA positive, 92.3%). VIA alone was found to yield a high false positive rate (86.2% *n* = 50/58). Of the VIA positive women, 77% were HR-HPV negative (Table 2).

**Table 2:** Baseline findings

	VIA+ HPV+ *n* = 10	VIA+ HPV- *n* = 58	VIA- HPV+ *n* = 26	Total *n* = 94
Age, mean (sd)	33.6 (2.9)	35.3 (5.9)	41.5 (7.9)	36.8 (6.9)
Biopsy (%)				
Benign	4 (40.0)	37 (63.8)	21 (80.8)	62 (66.0)
CIN1	2 (20.0)	13 (22.4)	4 (15.4)	19 (20.2)
CIN2	2 (20.0)	5 (8.6)	1 (3.8)	8 (8.5)
CIN3	1 (10.0)	3 (5.2)	0 (0.0)	4 (4.3)
ICC	1 (10.0)	0 (0.0)	0 (0.0)	1 (1.1)

Legend: *sd* standard deviation, *%* percentage, *VIA+* positive for visual inspection with acetic acid, *VIA* negative for visual inspection with acetic acid, *HPV+* positive test for human papillomavirus, *HPV* negative test for human papillomavirus, *CIN1,2,3* Cervical intra-epithelial neoplastic lesions grade 1,2,3, *ICC* invasive cervical cancer

Accuracy was assessed using a histological reference standard and the CIN classification described above. The results show that the static-image, Swede score assessment could correctly identify most CIN2+ lesions. In our study, an improvement in test accuracy is seen using a threshold of 4, where live versus static image assessment had a sensitivity of 76.9% (95% CI 46.2–95.0%) and 84.6% (95% CI 54.6–98.1%), respectively. The corresponding positive predictive values were found to be 90.9% (95% CI 75.7–98.1%) and 92.6% (95% CI 75.7–99.1%). Furthermore, Table 3 shows that different thresholds can yield a spectrum of accuracies. There were too few women with CIN3+ on biopsy to perform the test accuracy analysis on this severity of disease alone.

**Table 3:** Sensitivity and specificity in detecting CIN2+ for Live and Static examinations

Swede Score	Sensitivity	Specificity	PPV	NPV
	Live (95% CI)	Live (95% CI)	Live (95% CI)	Live (95% CI)
	Static (95% CI)	Static (95% CI)	Static (95% CI)	Static (95% CI)
**10**	0.0% (0.0–24.7%)	98.8% (93.3–100.0%)	86.0% (77.3–92.3%)	0.0% (0.0–97.5%)
	0.0% (0.0–24.7%)	100.0% (95.5–100.0%)	86.2% (77.5–92.4%)	NaN% (0.0–100.0%)
**9**	15.4% (1.9–45.4%)	98.8% (93.3–100.0%)	87.9% (79.4–93.8%)	66.7% (9.4–99.2%)
	0.0% (0.0–24.7%)	100.0% (95.5–100.0%)	86.2% (77.5–92.4%)	NaN% (0.0–100.0%)
**8**	30.8% (9.1–61.4%)	97.5% (91.4–99.7%)	89.8% (81.5–95.2%)	66.7% (22.3–95.7%)
	15.4% (1.9–45.4%)	95.1% (87.8–98.6%)	87.5% (78.7–93.6%)	33.3% (4.3–77.7%)
**7**	46.2% (19.2–74.9%)	88.9% (80.0–94.8%)	91.1% (82.6–96.4%)	40.0% (16.3–67.7%)
	53.8% (25.1–80.8%)	87.7% (78.5–93.9%)	92.2% (83.8–97.1%)	41.2% (18.4–67.1%)
**6**	53.8% (25.1–80.8%)	77.8% (67.2–86.3%)	91.3% (82.0–96.7%)	28.0% (12.1–49.4%)
	69.2% (38.6–90.9%)	67.9% (56.6–77.8%)	93.2% (83.5–98.1%)	25.7% (12.5–43.3%)
**5**	76.9% (46.2–95.0%)	39.5% (28.8–51.0%)	91.4% (76.9–98.2%)	16.9% (8.4–29.0%)
	76.9% (46.2–95.0%)	45.7% (34.6–57.1%)	92.5% (79.6–98.4%)	18.5% (9.3–31.4%)
**4**	**76.9% (46.2–95.0%)**	**37.0% (26.6–48.5%)**	**90.9% (75.7–98.1%)**	**16.4% (8.2–28.1%)**
	**84.6% (54.6–98.1%)**	**30.9% (21.1–42.1%)**	**92.6% (75.7–99.1%)**	**16.4% (8.5–27.5%)**
**3**	76.9% (46.2–95.0%)	37.0% (26.6–48.5%)	90.9% (75.7–98.1%)	16.4% (8.2–28.1%)
	84.6% (54.6–98.1%)	13.6% (7.0–23.0%)	84.6% (54.6–98.1%)	13.6% (7.0–23.0%)
**2**	76.9% (46.2–95.0%)	33.3% (23.2–44.7%)	90.0% (73.5–97.9%)	15.6% (7.8–26.9%)
	92.3% (64.0–99.8%)	8.6% (3.5–17.0%)	87.5% (47.3–99.7%)	14.0% (7.4–23.1%)
**1**	100.0% (75.3–100.0%)	2.5% (0.3–8.6%)	100.0% (15.8–100.0%)	14.1% (7.7–23.0%)
	92.3% (64.0–99.8%)	2.5% (0.3–8.6%)	66.7% (9.4–99.2%)	13.2% (7.0–21.9%)

Legend: *CI* confidence intervals

**Fig. 2:**
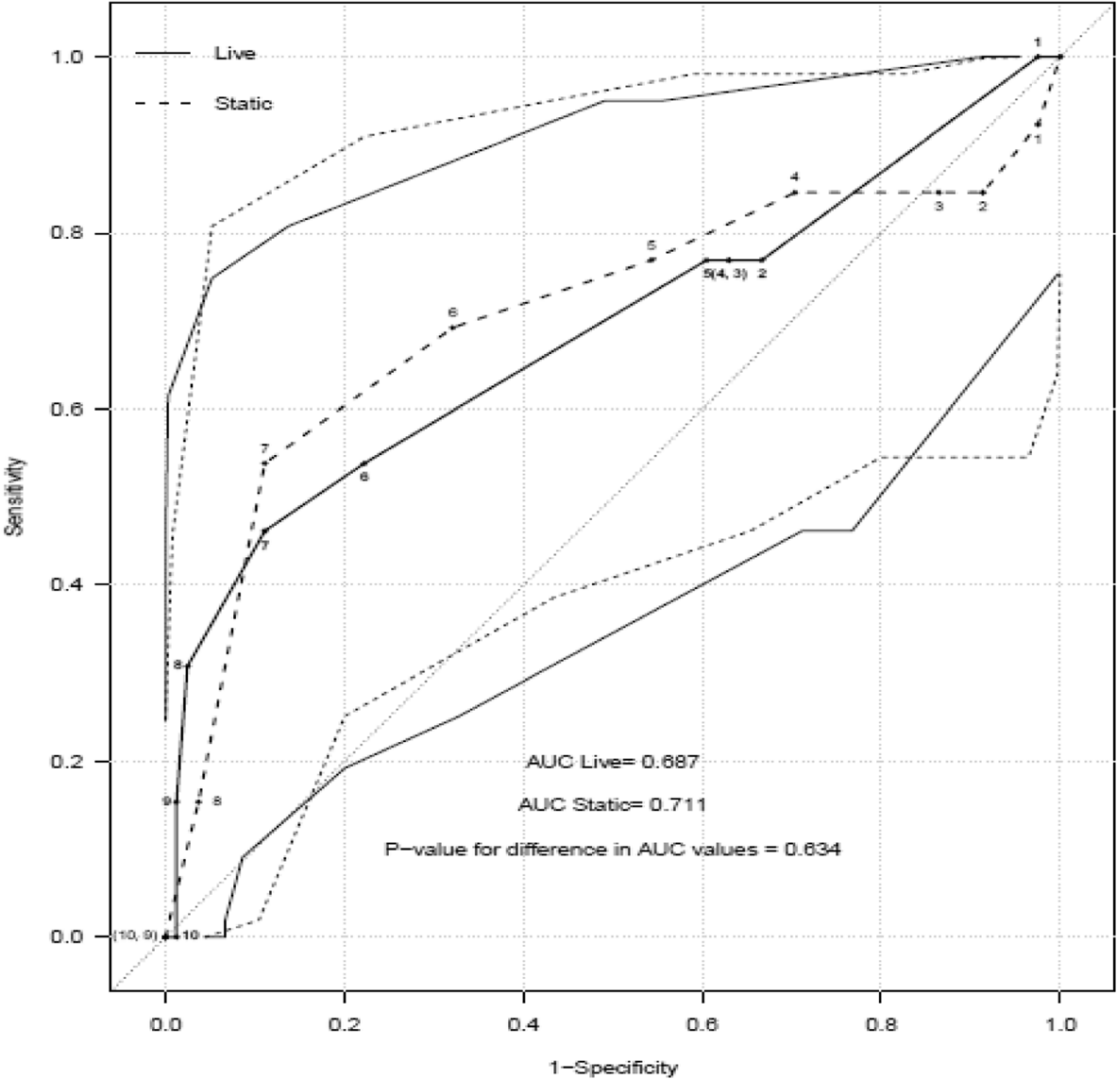
ROC curve and accuracy in detecting CIN2+ by Swede Score for live and static colposcopists. Bold line = test accuracy of each swede score when live assessment made, Dashed line = test accuracy at each swede score when using static images (average of all assessors). Lighter lines show the respective 95% confidence intervals. AUC = area under the curve

**Fig. 3:**
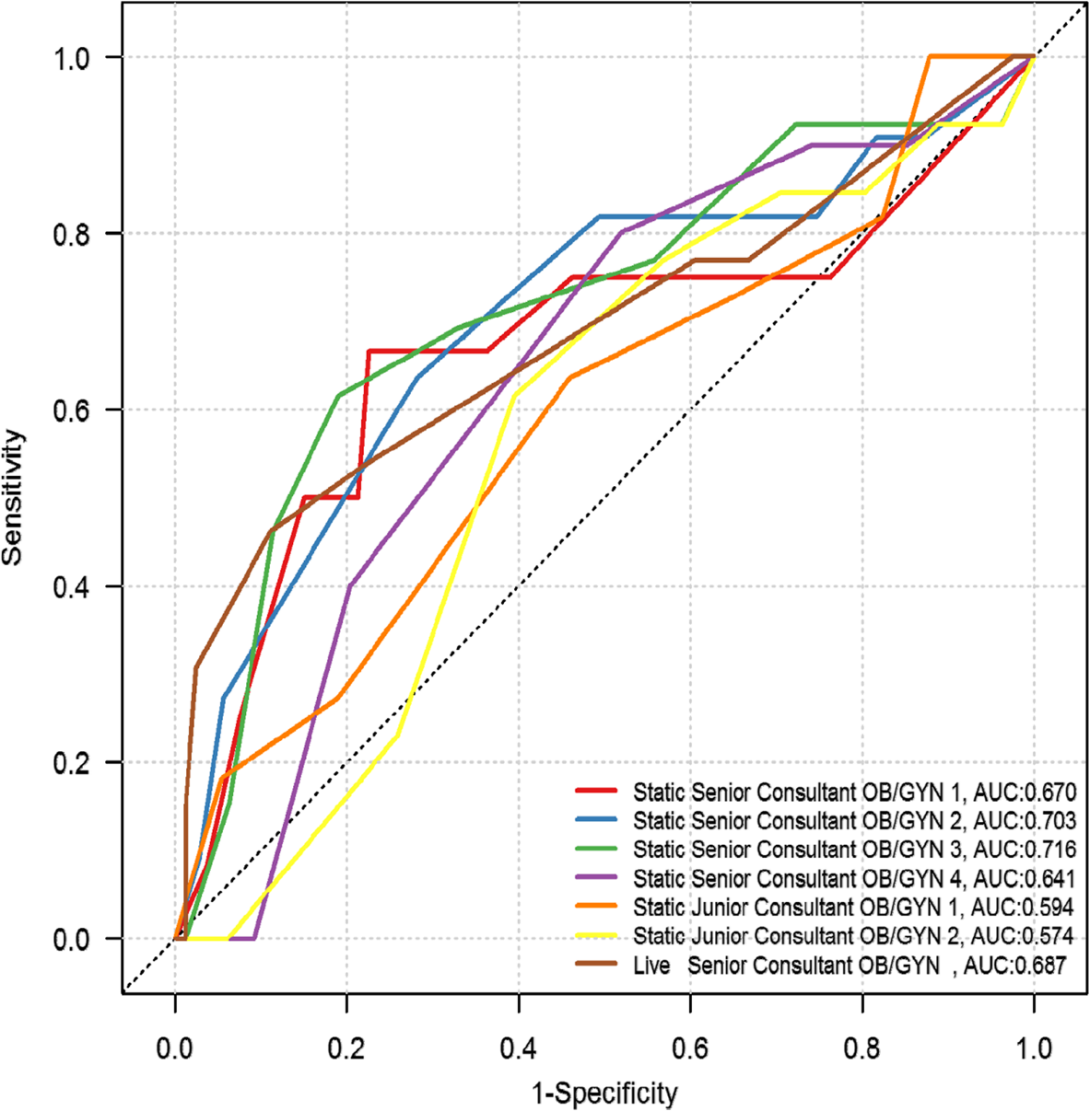
ROC curve of each colposcopist in detecting CIN2+ by Swede score. Each color depicts the assessment accuracy of one gynecologist, as per the key on the bottom right hand corner of the figure. AUC = area under the curve

Our findings showed that using static images are at least as good as live examination (Table 3) in colposcopic assessment of the cervix. The equivalence of live versus static examination was further illustrated in Figs. 2 and 3. There were no differences in the overall Area Under the Curve (AUC) value for live versus static assessments in detecting CIN2+ lesions (*p* = 0.63). The median number of images per patient was 5 (range 3–6). The majority of women (79.8%) had five or six images obtained at live-colposcopy. With an increased number of images to review, static image assessors were more accurate with their assessments (review of 5 images was associated with AUC = 0.690 and review of six images was associated with AUC = 0.775). There were too few positive biopsies in the groups of women who had three or four images taken to perform this analysis.

We found good agreement between both static and live assessors, measured by the weighted ĸ statistic (ĸ statistic > 0.6) among senior consultants. Fig. 3 shows the ROC for each colposcopist to detect CIN2 lesions by Swede score. One live colposcopist (Brown line) and six static image colposcopists (four of whom were expert colposcopists; Red, blue, green purple lines) and two junior (orange and yellow lines). The area under the curve score for the live colposcopy, performed by a gynecology oncologist, was 0.687. The AUC for the static image colposcopists ranged between 0.64–0.72. The results showed no difference in the detection of CIN2+ lesions between live and static assessors (AUC = 0.69 and 0.71, *p* = 0.63). Two static image colposcopists demonstrated even greater sensitivity than the expert live colposcopist (0.70 and 0.73 compared to 0.69). The two junior colposcopists achieved AUC of 0.57 and 0.59. Fig. 2 shows the summed average of all static colposcopists compared to the live colposcopist.

## Discussion

Our results found that CIN2+ lesions can be accurately diagnosed through static images using the Swede score. We also found that this method was as at least as effective as that achieved in a live assessment (AUC live = 0.69 and AUC static 0.71, p = 0.63).

A strength of the study is that cervical biopsy served as the reference standard and was performed on every patient. Biopsies were analyzed in a single site laboratory. A range of assessor experience was included in the analysis, adding to the generalizability of our findings. Assessments were standardized using the validated scoring tool, the Swede score [23, 27]. However, a notable limitation is that the quality of the monitors used to view the images and the image size viewed were not controlled for, which may have affected the assessment made. Timing of image capture after the application of solutions, and detailed instructions about the images to capture were not included and left to the discretion of the live colposcopist. The number of images per patient was not standardized which is considered a weakness of the study and no adjustment was made in the analysis for autocorrelation. Biopsies were taken of the worst lesion, although multiple biopsies may have improved detection of CIN2+ lesions. We also elected to use a histologically proven CIN2+ as the reference, because this has greater application in the real-world setting. Using the histological reference of CIN3 may have resulted in improved test accuracies and is also known to have better reproducibility in colposcopic examination. In addition, inter-observer variation is an inherent problem in colposcopy and is a characteristic limitation of studies such as this. We used Swede score in an effort to counteract the limitations of inter-observer variation in colposcopy [28–30]. The sample size is moderate in relation to other studies performed in this field, however, a larger sample size would give greater integrity to the findings.

Literature on the application of telemedicine in colposcopy remains divided [31]. Previous landmark papers using the ASCUC/LSIL triage study for cervical cancer (ALTS) data questioned the accuracy of static images in detecting borderline lesions [28, 29]. However, others have shown that the development of smartphones (not yet developed at the time of the above study) and static images can be a useful adjunct to colposcopy [8, 12, 32–34]. Gauthier et al. [12] performed a cross-sectional study using photographs (taken with 13 megapixels, autofocus and 2X optical zoom) as an adjunct to VIA and VILI. This study included 88 women, with one live colposcopist and four static colposcopists. They reported that live evaluation had a sensitivity of 28.6 (95% CI 3.7–71) whereas the best static evaluation had a sensitivity of 85.7 (95% CI 42.1–99.6). In an effort to address the issue of inter-observer variability, the live colposcopist also assessed the static images four months after the original assessment, which increased the sensitivity to 71.4 (95% CI 29–96.3). Further, Liu et al. [32] evaluated the largest number of static colposcopy images, where 558 women were assessed by six live colposcopists and one static image colposcopist. This study reported a 92% agreement between live and static assessments (ĸ statistic = 0.39 95% CI 0.21–0.57) using colposcopic signs including acetowhitening. Analysis of the ALTS data showed only fair correlation between evaluators (ĸ statistic 0.26, 95% CI 0.22–0.31). A subsequent analysis has also been performed with full representation of cervical precancerous lesions, including CIN2+ lesions, in which 112 images were assessed by two colposcopists and a total of 939 images reviewed [29]. This study found that colposcopists agreed on the diagnosis for only 56.8% of images and concluded that colposcopic diagnosis using static images is poorly reproducible. There were no details provided with regards to image-capturing technology used in these earlier studies [12].

The reported improvement in the evaluation of static images between earlier and later studies may be associated with the evolution of image-capturing technologies. Gauthier et al. also attributed the significant difference in assessments to the increased expertise of the static assessor [12]. Furthermore, assessment of static images has the advantage of allowing more time to analyse and compare images in detail, without compromising patient comfort, as well as the ability to increase image size and repeated review of previous images. Improved detection may also result from high pixel images which can be manipulated to zoom in on suspicious regions and transformation zone comparisons can be made immediately with the native cervix. Advances in imaging technology during the decade separating the works described above, as well as in the transfer and quality of images, impact the quality of interpretation of static images; therefore older literature may be less relevant today.

The problem of inter-observer variation may be secondary to colposcopists failing to identify characteristics of lesions consistently when assessing lesion grade. All the studies described above used different assessment criteria. Jermoimo et al. and Massad used use a verified scoring system (the Reid score) [28, 29] and the present study uses the Swede score. The Swede score supersedes the older Reid’s index, which excludes lesion size and scores acetowhiteness differently [23]. Using a scoring system ensures all features are consistently assessed and may reduce inter-observer variability [23, 35]. A recent study examining the efficacy of both Swede score and Reid’s score found that the sensitivity and specificity of the two assessment tools was very high (sensitivity: 100% vs 96.9%, specificity: 88.4% vs 95.3%, respectively) [35]. These are both highly sensitive and specific conditional upon the threshold used. Standardization of the colposcopic examination also makes the practice more accessible and allows practitioners with differing educational and clinical expertise to perform it with improved accuracy. The performance of the scoring system in this study is lower than that reported in the earlier literature [23, 35]. This may be explained by sample size bias between studies.

It is often suggested that static images fail to show the dynamic changes in response to uptake of acetic acid [32], as acetowhitening has been found to have higher replicability and correlation to severity of disease [36, 37]. Our study refutes this because no differences in overall assessment of static versus live assessment was found. Furthermore, advances in technology and imaging devices may allow short videos to be used in place of static images, which may further improve remote assessments.

Telemedicine offers the potential to improve cervical screening in remote and low-resource settings. However, there are two questions that require more robust investigation: 1) can diagnosis of CIN2+ lesions by static images yield adequate test accuracy?, 2) is static image assessment comparable to live colposcopic assessment?. Our study suggests colposcopy telemedicine may be a solution to a significant global issue however, larger studies are needed to confirm these findings.

## Conclusions

Our cross-sectional pilot study indicates that CIN2+ lesions can be reliably detected by Swede score evaluation of static colposcopy images. However, larger studies are needed to further develop the colposcopy telemedicine concept. Telemedicine may offer reliable guidance in the management of significant precancerous cervical lesions in areas where direct specialist examination or input is not available. It is also useful for quality control, which is often missing in low-resource settings and of upmost importance in visually based screening programs.

## Additional file

Additional file 1: License reprint Swede Score Model Agreement between University of Bern, Institute of Social and Preventative Medicine -- Katayoun Taghavi (“You”) and Wolters Kluwer Health, Inc. (“Wolters Kluwer Health, Inc.”) consisting of license details and the terms and conditions provided by Wolters Kluwer Health, Inc. and Copyright Clearance Center. (HTM 23 kb)

## Abbreviations

ALTS: ASCUC/LSIL triage study for cervical cancer
AUC: Area Under the Curve
CI: Confidence Intervals
CIN2 +: Cervical intra-epithelial neoplastic lesions greater than, or equal to grade 2 severity
CNCI: Chittaranjan National Cancer Institute
HR-HPV: High-risk oncogenic human papillomavirus
ĸ: kappa
LMIC: Low- and middle-income countries
ROC: Receiver operator curves
SD: Standard deviations
VIA: Visual Inspection with Acetic acid
WHO: World Health Organization
