# Supplementary material for: Colposcopy telemedicine: live versus static swede score and accuracy in detecting CIN2+, a cross-sectional pilot study
Source: BMC Womens Health. 2018 Jun 11;18:89. doi: 10.1186/s12905-018-0569-1 (PMC6040214; doi:10.1186/s12905-018-0569-1)
Supplement: Supplementary file 1 — License reprint Swede Score Model Agreement between University of Bern, Institute of Social and Preventative Medicine -- Katayoun Taghavi (“You”) and Wolters Kluwer Health, Inc. (“Wolters Kluwer Health, Inc.”) consisting of license details and the terms and conditions provided by Wolters Kluwer Health, Inc. and Copyright Clearance Center. (HTM 23 kb) [file 12905_2018_569_MOESM1_ESM.htm]

RightsLink Printable License


|  |  |
| --- | --- |
| WOLTERS KLUWER HEALTH, INC. LICENSE TERMS AND CONDITIONS | |
| Apr 24, 2018 | |
| ---  ---   This Agreement between University of Bern, Institute of Social and Preventative Medicine -- Katayoun Taghavi ("You") and Wolters Kluwer Health, Inc. ("Wolters Kluwer Health, Inc.") consists of your license details and the terms and conditions provided by Wolters Kluwer Health, Inc. and Copyright Clearance Center. | |
| **All payments must be made in full to CCC. For payment instructions, please see information listed at the bottom of this form.** | |
| License Number | 4334670778724 |
| License date | Apr 23, 2018 |
| Licensed Content Publisher | Wolters Kluwer Health, Inc. |
| Licensed Content Publication | Journal of Lower Genital Tract Disease |
| Licensed Content Title | The Swede Score: Evaluation of a Scoring System Designed to Improve the Predictive Value of Colposcopy |
| Licensed Content Author | Julie Bowring, Bjorn Strander, Martin Young, et al |
| Licensed Content Date | Oct 1, 2010 |
| Licensed Content Volume | 14 |
| Licensed Content Issue | 4 |
| Type of Use | Journal/Magazine |
| Requestor type | Academic/Educational | |
| Sponsorship | Commercial | |
| Format | Print and electronic | |
| Portion | Figures/table/illustration | |
| Number of figures/tables/illustrations | 1 | |
| Figures/tables/illustrations used | Swede Score Model | |
| Author of this Wolters Kluwer article | No | |
| Will you be translating? | No | |
| Title of the content | Swede Score Model | |
| Publication the new content is in | BMC Women's Health | |
| Publisher of your content | Springer Nature | |
| Author of the content | Katayoun Taghavi, Dipanwita Banerjee, Ranajit Mandal, Helena Kopp Kallner, Malin Thorsell, Therese Friis, Ljiljana.Kocoska-Maras, Björn Strander, Albert Singer, Elisabeth Wikström | |
| Expected publication date | May 2018 | |
| Estimated size of content (pages) | 1 | |
| Requestor Location | University of Bern, Institute of Social and Preventative Medicine Finkenhubelweg 11   Bern, Bern 3012 Switzerland Attn: University of Bern, Institute of Social and Preventative Medicine |
| Billing Type | Credit Card |
| Credit card info | Visa ending in 3253 |
| Credit card expiration | 06/2019 |
| Total | 96.47 CHF |
| Terms and Conditions | |
| ﻿  **Wolters Kluwer Terms and Conditions**   1. **Transfer of License:**    Wolters Kluwer hereby grants you a non-exclusive license to reproduce    this material for this purpose, and for no other use, subject to the    conditions herein. 2. **Credit Line:** will be    prominently placed and include: For books – the author(s), title of    book, editor, copyright holder, year of publication; For journals – the    author(s), title of article, title of journal, volume number, issue    number, inclusive pages and website URL to the journal page. 3. **Warranties:**    The requestor warrants that the material shall not be used in any    manner which may be considered derogatory to the title, content, or    authors of the material, or to Wolters Kluwer. 4. **Indemnity:**    You hereby indemnify and hold harmless Wolters Kluwer and their    respective officers, directors, employees and agents, from and against    any and all claims, costs, proceeding or demands arising out of your    unauthorized use of the Licensed Material. 5. **Geographical Scope:**    Permission granted is non-exclusive, and is valid throughout the world    in the English language and the languages specified in your original    request. 6. Wolters Kluwer cannot supply the requestor with the original artwork, electronic files or a "clean copy." 7. Permission    is valid if the borrowed material is original to a Wolters Kluwer    imprint (Lippincott-Raven Publishers, Williams & Wilkins, Lea &    Febiger, Harwal, Rapid Science, Little Brown & Company, Harper    & Row Medical, American Journal of Nursing Co, and Urban &    Schwarzenberg - English Language, Raven Press, Paul Hoeber, Springhouse,    Ovid). 8. **Termination of contract:** If you opt    not to use the material requested above please notify RightsLink or    Wolters Kluwer within 90 days of the original invoice date. 9. This    permission does not apply to images that are credited to publications    other than Wolters Kluwer books/journals or its Societies. For images    credited to non-Wolters Kluwer books or journals, you will need to    obtain permission from the source referenced in the figure or table    legend or credit line before making any use of the image(s) or table(s). 10. **Modifications:** With the exception of text     size or color, no Wolters Kluwer material is permitted to be modified or     adapted without publisher approval. 11. **Third party material:**     Adaptations are protected by copyright, so if you would like to reuse     material that we have adapted from another source, you will need not     only our permission, but the permission of the rights holder of the     original material. Similarly, if you want to reuse an adaptation of     original LWW content that appears in another publishers work, you will     need our permission and that of the next publisher. The adaptation     should be credited as follows: Adapted with permission from Wolters     Kluwer: Book author, title, year of publication or Journal name, article     author, title, reference citation, year of publication. Modifications     are permitted on an occasional basis only and permission must be sought     by Wolters Kluwer. 12. **Duration of the license:** Permission     is granted for a one-time use only within 12 months from the date of     this invoice. Rights herein do not apply to future reproductions,     editors, revisions, or other derivative works. Once the 12 - month term     has expired, permission to renew must be submitted in writing.     1. For        content reused in another journal or book, in print or electronic        format, the license is one-time use and lasts for the 1st edition of a        book or for the life of the edition in case of journals.     2. If your Permission Request is for use on a website (which is not a journal or a book), internet, intranet, or any publicly accessible site, you agree to remove the material from such site after 12 months or else renew your permission request. 13. **Contingent on payment:**     While you may exercise the rights licensed immediately upon issuance of     the license at the end of the licensing process for the transaction,     provided that you have disclosed complete and accurate details of your     proposed use, no license is finally effective unless and until full     payment is received from you (either by publisher or by CCC) as provided     in CCC's Billing and Payment terms and conditions. If full payment is     not received on a timely basis, then any license preliminarily granted     shall be deemed automatically revoked and shall be void as if never     granted. Further, in the event that you breach any of these terms and     conditions or any of CCC's Billing and Payment terms and conditions, the     license is automatically revoked and shall be void as if never granted.     Use of materials as described in a revoked license, as well as any use     of the materials beyond the scope of an unrevoked license, may     constitute copyright infringement and publisher reserves the right to     take any and all action to protect its copyright in the materials. 14. **Waived permission fee:**      If the permission fee for the requested use of our material has been     waived in this instance, please be advised that your future requests for     Wolters Kluwer materials may incur a fee. 15. **Service Description for Content Services:** Subject     to these terms of use, any terms set forth on the particular order, and     payment of the applicable fee, you may make the following uses of the     ordered materials:     1. **Content Rental:** You        may access and view a single electronic copy of the materials ordered        for the time period designated at the time the order is placed. Access        to the materials will be provided through a dedicated content viewer or        other portal, and access will be discontinued upon expiration of the        designated time period. An order for Content Rental does not include any        rights to print, download, save, create additional copies, to        distribute or to reuse in any way the full text or parts of the        materials.     2. **Content Purchase:** You may        access and download a single electronic copy of the materials ordered.        Copies will be provided by email or by such other means as publisher may        make available from time to time. An order for Content Purchase does        not include any rights to create additional copies or to distribute        copies of the materials.    **For Journals Only:**   1. Please note that articles in the **ahead-of-print stage**    of publication can be cited and the content may be re-used by including    the date of access and the unique DOI number. Any final changes in    manuscripts will be made at the time of print publication and will be    reflected in the final electronic version of the issue. Disclaimer:    Articles appearing in the Published Ahead-of-Print section have been    peer-reviewed and accepted for publication in the relevant journal and    posted online before print publication. Articles appearing as publish    ahead-of-print may contain statements, opinions, and information that    have errors in facts, figures, or interpretation. Accordingly, Wolters    Kluwer, the editors and authors and their respective employees are not    responsible or liable for the use of any such inaccurate or misleading    data, opinion or information contained in the articles in this section. 2. Where a journal is being published by a learned society, the details of that society must be included in the credit line.    1. **For Open Access journals:** The       following statement needs to be added when reprinting the material in       Open Access journals only: "promotional and commercial use of the       material in print, digital or mobile device format is prohibited without       the permission from the publisher Wolters Kluwer. Please contact permissions@lww.com for further information."    2. **Exceptions:** In case of reuse from **Diseases       of the Colon & Rectum, Plastic Reconstructive Surgery, The Green       Journal, Critical Care Medicine, Pediatric Critical Care Medicine, the       American Heart Association Publications and the American Academy of       Neurology** the following guideline applies: no drug/ trade name or logo can be included in the same page as the material re-used. 3. **Translations:** If    granted permissions to republish a full text article in another    language, Wolters Kluwer should be sent a copy of the translated PDF.    Please include disclaimer below on all translated copies:    1. ***Wolters       Kluwer and its Societies take no responsibility for the accuracy of the       translation from the published English original and are not liable for       any errors which may occur.*** 4. **Full Text Articles:** Reuse of full text articles in English is prohibited.    **STM Signatories Only:**   1. Any    permission granted for a particular edition will apply also to    subsequent editions and for editions in other languages, provided such    editions are for the work as a whole in situ and does not involve the    separate exploitation of the permitted illustrations or excerpts. Please    click here to view the STM guidelines.    **Other Terms and Conditions:**    v1.17 | |
| **Questions? customercare@copyright.com or +1-855-239-3415 (toll free in the US) or +1-978-646-2777.** | |
|  | |
| ---  --- | |
